# Shaping the WHO Sexual Health Assessment of Practices and Experiences questionnaire: a descriptive study on the real-world example from Portugal

**DOI:** 10.1080/26410397.2025.2554458

**Published:** 2025-09-01

**Authors:** Ana Luísa Patrão, Lianne Gonsalves, Vanessa Brizuela, Pedro Nobre

**Affiliations:** a Assistant Researcher, Center for Psychology at the University of Porto (CPUP), Faculty of Psychology and Education Sciences, University of Porto (FPCEUP), Porto, Portugal.; b Scientist, UNDP/UNFPA/UNICEF/WHO/World Bank Special Programme of Research Development and Research Training in Human Reproduction (HRP), Department of Sexual and Reproductive Health and Research, World Health Organization, Geneva, Switzerland; c Technical Officer, UNDP/UNFPA/UNICEF/WHO/World Bank Special Programme of Research Development and Research Training in Human Reproduction (HRP), Department of Sexual and Reproductive Health and Research, World Health Organization, Geneva, Switzerland; d Full Professor, Center for Psychology at the University of Porto (CPUP), Faculty of Psychology and Education Sciences, University of Porto (FPCEUP), Porto, Portugal

**Keywords:** WHO questionnaire, sexual and reproductive health, implementation in Portugal, sexual behaviours, sexual health promotion

## Abstract

Sexual health is a major dimension of global health and well-being. Yet, evidence regarding its assessment at a worldwide level is scarce. Most population-based studies are conducted in a limited number of countries from the Global North using specific measures that do not allow for country comparison. The World Health Organization (WHO) led a process to create a global survey called the Sexual Health Assessment of Practices and Experiences (SHAPE) to assess sexual practices and behaviours that impact on health. This article aims to describe the application and feasibility of this questionnaire in an extended real-world context. It presents the results of the implementation process in Portugal, the first country to use it with a nationally representative sample. This descriptive study was conducted between 14th June and 15th October 2023, involving a sample of 2,010 individuals (52% women) living in Portugal, aged 18–95 (mean = 49.6 years). 1,426 participants responded online and 584 by telephone. Overall response rate was 30.9% (79.5% online, 12.4% by telephone) and 94% of responses were valid. The original SHAPE questionnaire took 17.7 min to answer on average (16.6 min online and 20.3 min by telephone). Including module G (assessing sexual problems), average time was 29.2 minutes. The relatively short response time and choice of formats suggest this tool provides a comprehensive picture of sexual health. It is hoped that it will be widely used in different health and research contexts, to enhance the global evidence base for the development of policies that promote sexual health.

This article aims to describe the application and feasibility of this questionnaire in an extended real-world context.

## Plain language summary

Recognising the importance of assessing health behaviours related to sexual and reproductive health, a group led by experts from the World Health Organization (WHO) has created the SHAPE questionnaire. This tool aims to assess sexual practices and behaviours that have an impact on health. Portugal was the first country in the world to address this questionnaire to a representative sample of people. Our study investigated the implementation process. Data were collected from a sample of 2010 people of both sexes, using online and telephone methodology. The overall response rate was 30.9%. 94.7% of responses were valid. The average length of time taken to answer the original SHAPE questionnaire was 17.7 minutes. These results reinforce the quality and feasibility of using this tool which could be applied in different contexts. This would allow for innovative comparative studies.

## Résumé

La santé sexuelle est une dimension majeure de la santé et du bien-être dans le monde. Pourtant, les données concernant son évaluation à l’échelle mondiale sont rares. La plupart des études axées sur la population sont menées dans un nombre limité de pays du Nord, à l’aide de mesures spécifiques qui ne permettent pas de comparaison entre pays. L’Organisation mondiale de la Santé (OMS) a lancé un processus visant à créer une enquête mondiale appelée « Évaluation des pratiques et expériences en matière de santé sexuelle » (SHAPE) pour évaluer les pratiques et comportements sexuels qui influent sur la santé. Cet article vise à décrire l’application et la faisabilité de ce questionnaire dans un contexte réel élargi. Il présente les résultats de sa mise en œuvre au Portugal, premier pays à l’utiliser auprès d’un échantillon représentatif au niveau national. Cette étude descriptive a été réalisée entre le 14 juin et le 15 octobre 2023, auprès d’un échantillon de 2010 personnes (dont 52% de femmes) visant au Portugal, âgées de 18 à 95 ans (moyenne = 49,6 ans). 1426 participants ont répondu en ligne et 584 par téléphone. Le taux de réponse global était de 30,9% (79,5% en ligne, 12,4% par téléphone) et 94% des réponses étaient recevables. Il fallait en moyenne 17,7 min pour répondre au questionnaire SHAPE original (16,6 min en ligne et 20,3 min par téléphone). En cas d’inclusion du module G (évaluation des problèmes sexuels), le temps moyen était de 29,2 min. Le temps de réponse relativement court et le choix des formats suggèrent que cet outil fournit une image complète de la santé sexuelle. Il est à espérer qu’il sera largement utilisé dans différents contextes de santé et de recherche, afin d’enrichir la base de données mondiale pour l’élaboration de politiques qui favorisent la santé sexuelle.

## Resumen

La salud sexual es una dimensión importante de la salud y el bienestar a nivel mundial. Sin embargo, la evidencia sobre su evaluación a nivel mundial es escasa. La mayoría de los estudios de base poblacional son realizados en un número limitado de países del norte global utilizando medidas específicas que no permiten comparación entre países. La Organización Mundial de la Salud (OMS) dirigió un proceso para crear una encuesta mundial llamada la Evaluación de Prácticas y Experiencias de Salud Sexual (SHAPE, por sus siglas en inglés) para evaluar las prácticas y los comportamientos sexuales que afectan la salud. Este artículo pretende describir la aplicación y viabilidad de este cuestionario en un contexto extendido del mundo real. Presenta los resultados del proceso de aplicación en Portugal, el primer país en utilizarlo con una muestra representativa a nivel nacional. Este estudio descriptivo fue realizado entre el 14 de junio y el 15 de octubre de 2023, con una muestra de 2010 personas (52% mujeres) que vivían en Portugal, entre 18 y 95 años (media = 49,6 años). 1426 participantes respondieron en línea y 584 por teléfono. La tasa de respuesta general fue de 30,9% (79,5% en línea, 12,4% por teléfono) y 94% de las respuestas eran válidas. En promedio, llevó 17,7 minutos contestar el cuestionario SHAPE (16,6 minutos en línea y 20,3 minutos por teléfono). Incluyendo el módulo G (evaluación de problemas sexuales), el tiempo promedio fue de 29,2 minutos. El tiempo de respuesta relativamente corto y la elección de formatos indican que esta herramienta ofrece un panorama completo de la salud sexual. Se espera que se utilice ampliamente en diferentes contextos de salud y de investigación, para ampliar la base de evidencia mundial para la elaboración de políticas que promuevan la salud sexual.

## Introduction

Sexual health is a state of physical, emotional, mental, and social well-being regarding sexuality.^1^ Reproductive health is defined as a state of physical, mental, and social well-being in all that relates to the reproductive system, its functions, and processes, and provides for people to have a satisfying and safe sex life and the ability to reproduce, as well as the freedom to decide whether, when, and how often to do so.^2^ Both sexual and reproductive health (SRH) are important dimensions of overall health and well-being.^2^

Several studies have shown sexual intimacy has a significant role in people's lives and a buffering effect against adverse situations such as chronic stress, depression, and suicidal ideation.^1,3–6^ In addition, difficulties in sexual intimacy can be a source of stress that exacerbates existing problems.^7^ For these reasons, SRH has received a great deal of attention worldwide and with certain services (HIV prevention and treatment, safe pregnancy and delivery, contraception services) receiving substantial funding globally, with a focus on related health outcomes.^8–10^ However, behavioural issues of sexuality, which have a high impact on health, have been neglected.^8^

Recognising the importance of assessing health behaviours related to sexuality and reproduction in all their complexity, within a human rights framework, a group led by experts from the World Health Organization (WHO) has created a research tool, called the Sexual Health Assessment of Practices and Experiences (SHAPE) questionnaire, which aims to assess sexual practices and behaviours that have an impact on health.^8,11,12^ The development of this questionnaire began with a multi-stage consultative process,^13^ with the draft instrument subsequently tested and refined in waves through a 19-country cognitive testing study to assess the interpretability and comparability of the instrument in different contexts.^8,11^

In 2023, during the final wave of the multi-country study, a late-stage version of what would become the SHAPE instrument was used with a nationally representative sample in Portugal. This step was very important to understand the extent to which this instrument, which is intended to be global, is valid and feasible in real-world contexts. As sexual and reproductive health issues are a somewhat intimate and sensitive topic for the general population, it was extremely important to know all the details of the development of this first large-scale investigation with this instrument. Findings from this implementation helped shape the development of the final tool. This article aims to present the results of the process of implementing the tool in Portugal, the first country to do so with a nationally representative sample. Specifically, this article aims to describe how this questionnaire was applied in an extended real-world context and its feasibility.

## Methods

### Study design and population

The study is descriptive and has a representative sample of people (*n* = 2010) living in Portugal. This study is part of a larger project entitled “NEUROPSYCHSEX: Neuro-Psycho-Physiological Determinants of Sexual Health”, funded by the Portuguese Foundation for Science and Technology (April 2023 - end scheduled for July 2025), whose aim is to understand the biopsychosocial predictors of sexual health in the Portuguese population, as well as to create a diverse and inclusive online intervention for sexual difficulties.

### Data collection procedures

The present study was conducted through mixed data collection online and by telephone between 14th June and 15th October 2023. The participants were members of an online panel, selected by cross quotas according to gender, age group, and region (proportionally selected for each region of mainland Portugal and the Azores and Madeira islands). The inclusion criteria were as follows: living in Portugal, aged 18 or over, speaking Portuguese fluently, and having a landline and/or internet access.

Online data collection was carried out using a structured questionnaire with open and closed questions, made available on a customised platform adapted for this purpose. The participants responding to the questionnaire were part of an online panel made up of people from the Portuguese population who, at some point, had signed up to have access to online questionnaires, which they answered in exchange for an incentive (usually vouchers). There was no interaction between the interviewers and the participants in this data collection modality.

The telephone interviews were based on the same structured questionnaire used online, but in this modality, there was voice interaction between the interviewer and the participants. The (landline) telephone numbers were selected randomly from existing databases (public telephone directories). Since the telephone number is only associated with one region and a home, the respondent is selected based on the quotas, after someone answers the call. In other words, the person who answered the phone is asked if there is anyone in the household with the sociodemographic characteristics of the quotas. The questionnaire is only started once a respondent has been identified who fulfils these requirements and has consented to take part.

Data collection was carried out by a company specialising in population-based studies, with experienced interviewers trained in interview methods, who underwent specific training for this study (explanation of the objectives, analysis of each question, and simulation of interviews), as well as a fieldwork manager with experience in supervising and managing teams of interviewers.

### Variables

This study included sociodemographic variables to characterise the study population and variables related to the implementation process of the questionnaire. Sociodemographic variables included:

*Sex at birth*: assessed through the question “When you were born, were you considered:” with the answer options “male, female, another term (specify), and I prefer not to answer”. *Gender*: measured by the question “Today you identify yourself as:”, with the answer options “man/male, woman/female, another term (specify), and I prefer not to answer”.

*Sexual orientation*: assessed through the question “Do you think of yourself as:” with the answer options “gay or lesbian, heterosexual, bisexual, another form (identity) not listed here, I don't know, and I prefer not to answer”.

*Age*: Participants were asked about their age and, later, the variable was categorised into three age groups: 18–38, 40–64, and 65 and over.

*Marital status*: was asked the question “What is your marital status?”, with the answer options “single, married or in a stable union, separated but legally married, divorced, widowed, and prefer not to answer”.

*Education*: assessed through the question “What is your highest level of education?”, with the answer options “none, ≤4 years of schooling, ≤6 years of schooling, elementary school (9 years of schooling), high school (12 years of schooling), college/university, no answer”.

*Race/ethnicity*: measured by the question “Which of the following racial/ethnic groups do you consider yourself to belong to?”. The answer options were as follows: white/European origin, black/African descent, Asian/Asian origin, Roma, Multiracial, none, doesn't know, other, prefer not to answer.

Implementation process variables included:

*Data collection method*: collected by the data collection team using the “telephone” or “online” options.

*Response rate*: calculated using the percentage of respondents who completed the interview out of the total number of people contacted.

*Validity of responses*: all complete answers were considered valid; incomplete or responses stating “I prefer not to answer” were considered invalid responses.

*Response duration*: the response time was calculated in minutes and only for valid (complete) responses.

### Translation and adaptation of the questionnaire to Portuguese

A version of the tool (version 15) was made available by the WHO. The final version in English has been published and can be consulted.^8,11,12^ For the adaptation to Portugal, the research team was provided with a revised version of the tool (version 17) in its original language (English), together with a Brazilian Portuguese translation of that version from the Brazilian site of the WHO cognitive testing study. Using a specialised professional in the retroversion phase, adaptations were necessary for cultural reasons and linguistic meaning. In addition, between 5th and 13th June 2023, the tool was piloted with the participation of 40 people (different to the people who participated in the study), with male and female participants of different ages, from different regions of the country.^14^

The Portuguese version of the questionnaire included the six original modules from the tool (A–F), as well as an additional module (G): (A) sociodemographic and health data, (B) sexual health, (C) sexual biography, (D) sexual practices, (E) social perceptions and beliefs, (F) identity and rights, and (G) sexual problems, health and quality of life. The complete adaptation and translation process as well as the Portuguese version of the questionnaire have been published previously.^14^

### Reflexivity statement

Authors include three cisgender women and one cisgender man, and are psychologists and public health researchers with experience in sexual and reproductive health and rights research and policy. All the authors live in a European context (Portugal and Switzerland). Two of our team members were involved in the development and refinement of the tool that was used in this study. The other two are native Portuguese speakers, based in Portugal, and led the study’s implementation. We began this analysis with background and experience on the challenges faced by researchers and policymakers in collecting data on sexual practices and attitudes, including issues around sensitivities relating to sexual health and language. We also began this analysis with a deep commitment to making sexual health and well-being an integral component of population health.

### Data analysis

Descriptive statistics (relative and absolute frequencies) were calculated to characterise the sample according to the sociodemographic variables of interest (e.g. sex at birth, sex currently considered, age, sexual orientation, marital status, education, race/ethnicity). Chi-squared tests were carried out to assess different sociodemographic characteristics according to the data collection method (online and telephone). Descriptive statistics (median, mean, standard deviation, range) were calculated on the valid responses to the overall questionnaire and the different modules, analysing the total sample and the two data collection methods. Here, the proportion of questions with a valid answer (all except “I prefer not to answer”) to the total number of questions was calculated. Valid answers are those that refer to complete answers to the questionnaire, including when participants report that they prefer not to answer. To assess the differences in valid responses according to sociodemographic characteristics, a *t*-test was carried out for independent groups (sex) and ANOVAs for the remaining variables (age group, sexual orientation, marital status, education, race/ethnicity). To evaluate the average duration (in minutes) of responses to the modules (from A–G and from A–F), descriptive statistics (average) were carried out for the total and the different data collection methods. Finally, in order to assess the average response time according to specific sociodemographic characteristics, difference tests were carried out (*t*-test for independent groups, in this case sex; and ANOVAs for the other variables).

The IBM Statistical Package for Social Sciences (IBM SPSS for Windows version 27.0, IBM Corp, Armonk, NY, USA) software was used for all statistical analyses.

### Ethical considerations

As this is an intimate subject and may be sensitive for the general population, various ethical procedures were considered, particularly concerning the informed consent form. In the online version, participants could read the information and indicate their intention to participate, which was indicative of consent to participate. For the telephone interview, this information was read out, people gave their verbal agreement which the interviewers recorded. The information provided included the purpose of the study, as well as the total confidentiality of their identity and data, and that they were free to withdraw from participation at any time. Contact information for the team responsible for the study was included should participants want to reach out. The telephone conversations were not recorded. The study protocol was approved by the Ethics Committee of the Faculty of Psychology and Educational Sciences of the University of Porto (reference 2023/04-10), on 27 April 2023.

## Results

### Sociodemographic characteristics and data collection method

In total, 2010 people completed the questionnaire, corresponding to a sampling error of 2% in a random sampling process, for a 95% confidence level. Of these, 52.3% were women. The average age was 49.6 (SD = 18.9; Range: 18–95). More details on the characteristics of the sample can be seen in Table 1. Of the participants, 1426 responded online and 584 through telephone interviews.

**Table 1.** Main sociodemographic characteristics of the participants (*N* = 2010)

Sociodemographic characteristics	*n*	%
**Sex at birth**		
Male	959	47.7
Female	1051	52.3
**Gender**		
Male	962	47.9
Female	1045	52.0
Other	3	0.1
**Sexual orientation**		
Heterosexual	1840	91.5
Bisexual	84	4.2
Gay or lesbian	29	1.4
Another identity not listed	17	0.8
Doesn't know	19	0.9
Prefer not to answer	21	1.2
**Age group**		
18–39 years	577	28.7
40–64 years	879	43.7
≥65 years	554	27.6
**Marital status**		
Single	491	24.4
Married or in a stable union	1196	59.5
Separated but legally married	23	1.2
Divorced	175	8.7
Widowed	100	5.0
Prefer not to answer	25	1.2
**Education**		
None	12	0.6
≤4 years of schooling	184	9.2
≤6 years of schooling	150	7.5
Elementary school (9 years of schooling)	208	10.3
High school (12 years of schooling)	643	32.0
College/university	806	40.1
No answer	7	0.3
**Race/ethnicity**		
White/European origin	1872	93.1
Black/African descent	32	1.6
Asian/Asian origin	7	0.3
Roma	4	0.2
Multiracial	46	2.3
None	10	0.5
Doesn't know	9	0.4
Other	12	0.7
Prefer not to answer	18	0.9

As can be seen in Table 2, the responses for each type of modality varied according to some of the participants’ sociodemographic characteristics. More men than women responded to the online modality (51.6% vs 48.4%), and significantly more women than men responded to the telephone modality (61.8% vs 38.2%). In addition, the online modality included a greater number of bisexual, gay, and lesbian respondents (1.8% vs 0.5%). Telephone had a higher proportion of older people than online. Regarding marital status, the majority of the participants in both modalities were married or in a stable union (61.6% and 54.3%), the ones who responded by telephone presented a higher percentage of widowed individuals (14% vs 1.3%). Concerning education, in the online modality, the majority of participants had a university degree (52.2%), while in the telephone modality, the highest percentage (31%) was among those with four or fewer years of schooling. Regarding ethnicity, in both modalities most participants considered themselves to be white/European origin, although the online sample had a higher percentage of other ethnicities (e.g. multiracial).

**Table 2.** Participation by data collection method according to selected sociodemographic characteristics (*N* = 2010)

Sociodemographic characteristics	Total (*N* = 2010) %	Online (*n* = 1426) %	Phone (*n* = 584) %	*p-value
**Sex at birth** Male	47.7	51.6	38.2	<0.001
Female	52.3	48.4	61.8	
**Gender**				< 0.001
Man	47.9	51.7	38.5	
Woman	52	48.2	61.3	
Prefer not to answer	0.1	0.1	0.2	
**Sexual orientation**				< 0.001
Gay or lesbian	1.4	1.8	0.5	
Heterosexual	91.5	89.6	96.2	
Bisexual	4.2	5.5	0.9	
Another identity not listed	0.8	1.1	0.2	
Doesn't know	0.9	1.1	0.7	
Prefer not to answer	1.0	0.8	1.5	
**Age group**				< 0.001
18–39 years	28.7	34.2	15.2	
40–64 years	43.7	50.0	28.4	
≥65 years	27.6	15.8	56.3	
**Marital status**				< 0.001
Single	24.4	25.6	21.6	
Married or in a stable union	59.5	61.6	54.3	
Separated but legally married or in a stable union	1.1	1.6	0.0	
Divorced	8.7	9.4	7.0	
Widowed	5.0	1.3	14.0	
Prefer not to answer	1.2	0.5	3.1	
**Education**				< 0.001
None	0.6	0.1	1.7	
≤4 years of schooling	9.2	0.9	29.3	
≤6 years of schooling	7.5	1.2	22.8	
Elementary school (9 years of schooling)	10.3	4.7	24.1	
High school (12 years of schooling)	32	40.8	10.4	
College/university	40.1	52.2	10.4	
No answer	0.3	0.0	1.2	
**Race/ethnicity**				< 0.001
White/European origin	93.1	92.5	94.7	
Black/African descent	1.6	1.5	1.7	
Asian/Asian origin	0.3	0.5	0.0	
Roma	0.2	0.1	0.3	
Multiracial	2.3	2.7	1.2	
None	0.5	0.6	0.2	
Don’t know	0.4	0.5	0.3	
Other	0.6	0.7	0.3	
Prefer not to answer	0.9	0.8	1.2	

*Chi-square test.

### Response rate

The overall response rate was 30.9%. Specifically, among those who responded to the online survey, the response rate was 79.5%, and among those who responded by telephone, the response rate was 12.4%.

### Validity of responses

According to the sociodemographic characteristics, the lowest averages in valid responses (even though they are high) were observed in women, older people, those who preferred not to answer about their sexual orientation, those with lower or no education, widows/widowers, and those who preferred not to answer about their sexual orientation and race/ethnicity (see Table 3).

**Table 3.** Proportion of valid responses by sociodemographic characteristics

Sociodemographic characteristics	Median %	Mean %	*Difference test
**Gender**			<0.001
Male	98.2	95.4	
Female	98.2	94.0	
**Age group**			<0.001
18–39 years	98.6	96.1	
40–64 years	98.2	95.4	
≥65 years	97.7	92.0	
**Sexual orientation**			<0.001
Heterosexual	98.2	94.9	
Bisexual	98.6	96.8	
Gay or lesbian	98.2	95.7	
Another identity not listed	98.2	96.1	
Doesn't know	97.7	94.3	
Prefer not to answer	59.7	61.7	
**Marital status**			<0.001
Single	98.6	95.9	
Married or in a stable union	98.2	94.7	
Separated but legally married or in a stable union	97.3	95.6	
Divorced	98.2	95.4	
Widowed	97.7	91.6	
Prefer not to answer	83.3	71.6	
**Education**			<0.001
None	92.5	89.8	
≤4 years of schooling	97.3	91.0	
≤6 years of schooling	98.6	94.1	
Elementary school (9 years of schooling)	97.7	93.5	
High school (12 years of schooling)	98.2	95.0	
College/university	98.6	96.2	
Prefer not to answer	45.2	41.8	
**Race/ethnicity**			<0.001
White/European origin	98.2	94.9	
Black/African descent	97.7	93.1	
Asian/Asian origin	98.2	97.3	
Roma	96.2	96.5	
Multiracial	99.3	96.0	
None	97.5	93.3	
Don’t know	92.3	89.3	
Other	98.6	97.2	
Prefer not to answer	63.3	65.2	

*Difference test: *t*-test for sex; ANOVAs for the other variable.

Table 4 shows the results of the valid responses for each module, according to the data collection method (and overall). Overall, 94.7% of responses were valid. Considering the average values, the lowest values are for module G, which corresponds to questions about sexual problems. However, it is important to emphasise that uptake was lower in the telephone method. The modules with the highest uptake were A (sociodemographic and health data) and F (identity and rights) for the total and both types of modality response.

**Table 4.** Proportion of valid responses for each module by data collection method

		Complete Questionnaire	Module A	Module B	Module C	Module D	Module E	Module F	Module G
**Total**	**Median**	98.2%	100%	100%	100%	100%	100%	100%	97.7%
	**Mean**	94.7%	99.4%	98.3%	98.2%	95.8%	96.8%	99.4%	89.4%
	**Standard Deviation**	8.9%	3.5%	5.2%	7.0%	11.8%	10.9%	2.7%	18.9%
	**Minimum**	30.3%	33.3%	60%	40%	17.4%	0%	64.7%	0%
	**Maximum**	100%	100%	100%	100%	100%	100%	100%	100%
**Online**	**Median**	98.2%	100%	100%	100%	100%	100%	100%	97.7%
	**Mean**	96%	99.3%	98.9%	97.9%	97.1%	97.1%	99.3%	92.3%
	**Standard Deviation**	7.2%	3.3%	4.1%	8.0%	10.4%	10.5%	2.4%	14.8%
	**Minimum**	30.3%	55.6%	70%	40%	17.4%	0%	64.7%	0%
	**Maximum**	100%	100%	100%	100%	100%	100%	100%	100%
**Phone**	**Median**	97.7%	100%	100%	100%	100%	100%	100%	98.9%
	**Mean**	91.4%	99.4%	96.7%	98.9%	92.8%	96.3%	99.4%	82.5%
	**Standard Deviation**	11.5%	3.8%	6.9%	3.4%	14.4%	11.8%	3.2%	24.9%
	**Minimum**	31.7%	33.3%	60%	60%	21.7%	0%	70.6%	0%
	**Maximum**	100%	100%	100%	100%	100%	100%	100%	100%

Considering the incomplete questionnaires (*n* = 341), when checking the moments in the questionnaire where participants gave up, it remains relevant to point out question G1 (the first question after the end of module F: “In the last 6 months, have you experienced frequent or recurrent difficulties in experiencing satisfying sexual activities that cause you significant emotional distress or discomfort”) as the one where the most participants stopped answering (*n* = 140).

### Response duration

The average length of time taken to answer the full questionnaire, from modules A to G (the latter exists only in the Portuguese version) was 29.2 minutes, whereby the online version took an average 27.4 minutes, and the telephone version, 33.5 minutes. When considering the six modules of the original questionnaire (modules A–F) the average response time was 17.7, 16.6 minutes online, and 20.3 minutes on the phone.

When we cross-reference these data with the level of education and age, we see that older people took longer to respond to the questionnaire (21.3 minutes), and the most educated responded faster (16.1 minutes). There were no differences in response duration between men and women.

## Discussion

This study aimed to present the results of the implementation of a WHO tool on sexual behaviours and practices by assessing data collection method, response rate, validity of responses, and response duration in a real-life context in Portugal. Overall, we found that most people were able to respond to the questionnaire fully, with increased attrition with regard to questions on sexual problems, health, and quality of life satisfaction. These results have demonstrated that this instrument is a useful resource to help understand sexual and reproductive health in Portugal, which also encourages its use in other contexts around the world.

The response rate was very high for the online survey and lower for the phone interview. Our lower response rate for the telephone modality is in line with other investigations and their results.^15^ We found that participants who agreed to complete the survey by telephone were significantly older than those who responded online, which is in line with other studies.^16^ It is important to emphasise that the online responses were self-administered, and the telephone responses – where participation was lower – were recorded by the interviewer. We therefore consider it important to highlight the advantages and disadvantages of the different types of modalities. Online interviewing allows for greater privacy and requires fewer costs and resources in data collection. However, online literacy should be assessed among the older population to ensure that they are not excluded, which can be overcome by using the telephone modality. And, if possible and necessary, the face-to-face modality should also be an option for this type of study.^12^

The survey took on average less than 30 minutes to complete in the full version and less than 20 minutes in WHO’s original version. Although here too there was a discrepancy between the online and telephone modes, the average duration of both collection methods is adequate for this type of longer questionnaire.^16^ This is important because it informs the feasibility of this questionnaire (in part or in full) to be integrated into wider national and global health surveys.

During the pre-test (which had similar results), discomfort at talking to someone about such intimate topics was cited as the main reason for refusing to participate.^14^ It is important to note that the older age and lower educational level of telephone participants may have contributed to the discomfort cited. Even so, among those who agreed to participate, the completion rate was high for both groups and for the ones who completed the survey, the percentage of non-valid responses was very low. In addition, the overall acceptance of the questions was very high, and the modules considered most sensitive were answered by almost all the participants in the study. These results are encouraging and confirm that, despite the sensitive and intimate nature of the topic, the guarantee of confidentiality and privacy of the participants that is facilitated using online questionnaires, as well as the use of informed consent, enhances people's participation.^17^ It is also important to emphasise that there were always sensitive introductions to these more delicate issues. For example, when it came to issues of violence, we always made it clear that people could choose not to answer and move on if they felt uncomfortable talking about it, as well as seek help through the team. In addition, at the beginning of module G (the point where people gave up answering the most) on sexual dysfunction and distress, we made it clear that this section would talk about difficulties in intimacy, that the responses would be recorded and read without any judgement, that the aim was to understand the experience in an attempt to improve this field of study, and that they could seek help from the team if the questions made them feel uncomfortable.

When asked appropriately and sensitively, health surveys can include questions about people's sexual health. The assumption that a general population will not respond to sex and sexuality related questions does not hold. Rather, as was seen in the broader cognitive interviewing study to refine the SHAPE instrument, respondents may see these issues as an opportunity to talk about topics that are not normally addressed. This reality must be taken into account in global health contexts – not just those aimed at sexual health – as well as in health promotion policies.^11^

This study has strengths that are worth emphasising. The sample was large and representative of the Portugal population (except regarding education). In this context, it is important to emphasise that in terms of sexual orientation, our data are very similar to those found in other recent studies. According to a 30-Country Ipsos Global Advisor Survey,^18^ in Portugal, 6% of the population consider themselves to be non-heterosexual. In this study, the sum of people who consider themselves to be bisexual, gay or lesbian, and another identity not listed, is a little higher than that. These results reinforce the representativeness of the sample, although sexual orientation was not one of the variables considered in this representativeness at the outset. Therefore, this study also had the advantage of reaching out to groups that are not always represented in population studies in Portugal, such LGBTIQ+ persons, in a subject where inclusion and representativeness are essential. We also managed to get older people (some over 80 and 90) to respond fully to the questionnaire and to a topic that is not always accessible to this group. A population study that successfully includes LGBTIQ+ persons and older adults is vital for ensuring comprehensive, inclusive data. These groups are often underrepresented in research, leading to gaps in health care, social policy, and support services. By capturing their unique experiences and needs, such a study promotes equity, reveals disparities, and guides better resource allocation. It also challenges stereotypes, validates diverse identities, and informs interventions tailored to aging LGBTIQ+ populations. Ultimately, this inclusivity strengthens scientific accuracy and societal understanding, helping to create policies that reflect the true diversity of the population.

This is the first study to assess the real-world implementation of a sexual health instrument with a largely representative sample from Portugal. However, this study has some limitations, such as social desirability, especially in the telephone component, and self-reporting bias with the aspects of memory failure and discomfort in talking about an intimate subject. In addition, and despite being a largely representative study of the Portuguese population, its results cannot be fully generalised, because there are important issues, such as educational level, which were not representative of the total population. This is a study that cannot be compared with other similar studies, as it is the first to be developed in sexual health, with a representative sample, in Portugal. Additionally, although other countries are implementing SHAPE, this is the first study to do so with a nationally representative sample. Therefore, it is encouraged that in the future comparisons can be made between these results and similar studies.

**Table 5.** Average length (in minutes) of the questionnaire according to sex, age, and education. Modules from A to F (SHAPE original version)

Characteristics	Mean %	* Difference test
**Gender**		< .764
Male	17.8	
Female	17.6	
**Age group**		< .001
18-39 years	15.0	
40-64 years	17.2	
≥65 years	21.3	
**Education**		<.001
None	17.3	
≤4 years of schooling	20.6	
≤6 years of schooling	21.1	
Elementary school (9 years of schooling)	20.0	
High school (12 years of schooling)	17.3	
College/university	16.1	
Prefer not to answer	8.8	

*Difference test: *t*-test for sex; ANOVAs for the other variables.

The attempt to construct and adapt sexual and reproductive health questionnaires so that they can be used globally is a major challenge in terms of research and fieldwork. However, it is a worthwhile endeavour because it offers enormous possibilities for comparative and comprehensive studies in an area so lacking in evidence-based on population studies.^8^ Comparative studies, which are only possible using the same instruments and analyses, are the only ones capable of evaluating the relationship between local policies and their impacts on SRH, making it possible to identify the actions that achieve the most positive results.^19^ In addition, research in a single context, be it a country or a similar region, is not able to give a broad overview of global and regional trends (and solutions) to SRH challenges.^19,20^

## Conclusion

The results of the implementation of WHO’s tool in Portugal are encouraging and reinforce the quality and potential of this tool. The existence of a questionnaire that predominantly addresses the more behavioural and psychosocial components of SRH adapted to the Portuguese population and sensitive to its social and cultural particularities, but which at the same time can be used globally while maintaining its essential consistency, allows for the development of comprehensive and comparative studies that will contribute to the promotion of health in general, and sexual and reproductive health in particular. It is therefore hoped that this tool, which underwent a rigorous implementation and whose first application globally at a population level was in Portugal, will be widely used in different health and research contexts, allowing for greater knowledge of the Portuguese and global reality in terms of SRH and the development of evidence-based public policies.

## Author contributions

*Conceptualisation: ALP, PN. Formal analysis: ALP. Investigation: ALP, PN. Methodology: ALP, LG, VB, PN. Project administration: PN. Supervision: PN. Validation: PN, LG, VB. Writing – original draft: ALP. Writing – review & editing: ALP, LG, VB, PN. All authors approved the submission of the ﬁnal manuscript.*

## Provenance statement

This article was not commissioned and went through external peer review.
